# MicroRNAs and histone deacetylase inhibition-mediated protection against inflammatory β-cell damage

**DOI:** 10.1371/journal.pone.0203713

**Published:** 2018-09-27

**Authors:** Anna Lindeløv Vestergaard, Claus Heiner Bang-Berthelsen, Tina Fløyel, Jonathan Lucien Stahl, Lisa Christen, Farzaneh Taheri Sotudeh, Peter de Hemmer Horskjær, Klaus Stensgaard Frederiksen, Frida Greek Kofod, Christine Bruun, Lukas Adrian Berchtold, Joachim Størling, Romano Regazzi, Simranjeet Kaur, Flemming Pociot, Thomas Mandrup-Poulsen

**Affiliations:** 1 Department of Biomedical Sciences, University of Copenhagen, Copenhagen, Denmark; 2 Center for Non-coding RNA in Technology and Health, Department of Pediatrics, Herlev and Gentofte Hospital, Herlev, Denmark; 3 Steno Diabetes Center Copenhagen, Gentofte, Denmark; 4 Department of GLP-1 and T2D Biology, Novo Nordisk, Måløv, Denmark; 5 Hagedorn Research Institute, Gentofte, Denmark; 6 Copenhagen Diabetes Research Center, Department of Pediatrics, University Hospital Herlev, Herlev, Denmark; 7 Department of Fundamental Neurosciences, University of Lausanne, Lausanne, Switzerland; Institut de Pharmacologie Moleculaire et Cellulaire, FRANCE

## Abstract

Inflammatory β-cell failure contributes to type 1 and type 2 diabetes pathogenesis. Pro-inflammatory cytokines cause β-cell dysfunction and apoptosis, and lysine deacetylase inhibitors (KDACi) prevent β-cell failure *in vitro* and *in vivo*, in part by reducing NF-κB transcriptional activity. We investigated the hypothesis that the protective effect of KDACi involves transcriptional regulation of microRNAs (miRs), potential new targets in diabetes treatment. Insulin-producing INS1 cells were cultured with or without the broad-spectrum KDACi Givinostat, prior to exposure to the pro-inflammatory cytokines IL-1β and IFN-γ for 6 h or 24 h, and miR expression was profiled with miR array. Thirteen miRs (miR-7a-2-3p, miR-29c-3p, miR-96-5p, miR-101a-3p, miR-140-5p, miR-146a-5p, miR-146b-5p, miR-340-5p, miR-384-5p, miR-455-5p, miR-466b-2-3p, miR-652-5p, and miR-3584-5p) were regulated by both cytokines and Givinostat, and nine were examined by qRT-PCR. miR-146a-5p was strongly regulated by cytokines and KDACi and was analyzed further. miR-146a-5p expression was induced by cytokines in rat and human islets. Cytokine-induced miR-146a-5p expression was specific for INS1 and β-TC3 cells, whereas α-TC1 cells exhibited a higher basal expression. Transfection of INS1 cells with miR-146a-5p reduced cytokine signaling, including the activity of NF-κB and iNOS promoters, as well as NO production and protein levels of iNOS and its own direct targets TNF receptor associated factor 6 (TRAF6) and interleukin-1 receptor-associated kinase 1 (IRAK1). miR-146a-5p was elevated in the pancreas of diabetes-prone BB-DP rats at diabetes onset, suggesting that miR-146a-5p could play a role in type 1 diabetes development. The miR array of cytokine-exposed INS1 cells rescued by KDACi revealed several other miRs potentially involved in cytokine-induced β-cell apoptosis, demonstrating the strength of this approach.

## Introduction

Reduction of functional β-cell mass is a feature of both type 1 and type 2 diabetes (T1D and T2D, respectively), and inflammatory mechanisms including pro-inflammatory cytokines have been implicated as mediators of β-cell apoptosis in both disorders [1–3]. The pro-inflammatory cytokines interleukin-1β (IL-1β), interferon-γ (IFN-γ) and tumor necrosis factor-α (TNF-α) in synergy cause selective β-cell destruction *in vitro* [4–6]. The process involves endoplasmic reticulum, and mitochondrial and oxidative stress-induced apoptosis [7, 8] dependent on activation of mitogen activated protein kinases (MAPK) and the nuclear factor kappa B (NF-κB) transcription factor [9–11]. However, the exact mechanisms behind cytokine-induced β-cell death are not fully understood.

Cytokine-induced β-cell apoptosis requires active gene expression and protein translation [11]. We recently discovered that oral inhibitors of lysine deacetylases (KDACs), proven to be effective and safe in other inflammatory disorders such as systemic onset juvenile idiopathic arthritis [12] and graft-versus-host disease [13], prevent cytokine-induced β-cell apoptosis [14–19]. KDACs are enzymes that regulate gene expression and protein activity by deacetylating histone proteins, transcription factors, kinases, and other proteins [20, 21]. We found that all 11 classical KDACs are expressed and differentially regulated in β-cells, and that the β-cell protective effect of broad KDACi *in vitro* and *in vivo* was mainly conferred by inhibition of histone deacetylases 1 and 3 (HDAC1 and HDAC3) [15, 18, 19]. The protection was not associated with upregulation of gene expression as expected from the conventional concept that histone hyperacetylation leads to a more open chromatin structure accessible to the transcriptional machinery, but with downregulation of inflammatory gene expression [18]. KDACi caused hyperacetylation and thereby reduced NF-κB binding to inflammatory promoters, in part providing a molecular mechanism of action [14]. However, an additional mechanism could be hyperacetylation of histones upregulating expression of anti-apoptotic microRNAs (miRs). These in turn could act by e.g. repressing the translation of proteins that promote β-cell death via activation of the intrinsic (mitochondrial) death pathway.

miRs are small conserved non-coding RNAs that regulate translation and stability of specific target mRNAs [22]. miRs have been associated with a number of biological processes such as organ development, maturation and apoptosis in the immune system [23]. Importantly, several studies have implicated miRs in β-cell biology, diabetes, insulin resistance, and inflammation [24, 25]. We therefore aimed to identify miRs involved in cytokine-induced β-cell apoptosis by profiling miR regulation in insulin-producing cells exposed to inflammatory stress in the absence and presence of KDACi and validating the functional importance of candidate miRs in insulin-secreting cells and rodent and human islets, as moderating such miRs could be new promising approaches to future diabetes treatments.

## Materials and methods

### Cell culture

The rat insulinoma-derived β-cell line INS1 and the INSrαβ cell line, a maturation model with inducible pancreatic duodenal homeobox-1 (Pdx-1) expression, were generous gifts from Claes Wollheim and Pierre Maechler (University of Geneva, Switzerland). α-TC1, β-TC3 and INS1 cells were maintained in RPMI-1640 medium with GlutaMAX (Life Technologies), supplemented with 10% fetal bovine serum (FBS, Life technologies), 100 U/ml penicillin and 100 μg/ml streptomycin (1% P/S, Life Technologies), and 50 μM β-mercaptoethanol (Life Technologies). The cells were cultured at 37°C in a humidified atmosphere containing 5% CO_2_. Changing medium and passaging of cells were performed weekly. Studies with the INSrαβ cell line were performed as described previously [26]. All cells were *Mycoplasma* negative.

### Rat islets and pancreas

Islets were isolated from 3 to 6 day-old neonatal Wistar rat pups (Taconic, Ry, Denmark). The islets were cultured in RPMI-1640 medium with GlutaMAX (Invitrogen) supplemented with 1% P/S and 10% newborn calf serum (Invitrogen). Islets were transferred to new medium and 100 randomly picked islets per well were placed in 12-well plates and exposed for 2, 6 or 24 h to IL-1β (160 pg/ml, Sigma) alone or a mixture of IFN-γ (5 ng/ml, BD-Pharm) and IL-1β (160 pg/ml, Sigma).

BB-DP rats (M&B, Ll. Skensved, Denmark) were housed in a specific pathogen-free environment under controlled conditions of light and temperature with unlimited access to food and water [27]. The rats were weighed and blood glucose (BG) was measured thrice weekly. When BG was higher than 14 mmol/l it was checked the next day, and diabetes defined as BG > 14 mmol/l on two consecutive days. The rats were sacrificed at 37, 53, 80 or 120 days of age or at onset of diabetes. The mean age of diabetes onset was 81 days of age. Immediately after sacrifice, the pancreas was removed, snap-frozen and stored at -80°C until RNA extraction (n = 3–6 in each group).

Animal experiments, including anesthesia, were performed according with Danish veterinarian guidelines for animal care and welfare and were approved by the Danish Animal Experiment Inspectorate. No survival surgeries were performed. An IACUC (Institutional Animal Care and Use Committee) supervised all animal procedures. All principal decisions concerning the Animal Care and Use Programme in the mentioned fora are worked into Standard Operating Procedures. The Department’s animal care and use programme is accredited by AAALAC (Association for Assessment and Accreditation of Laboratory Animal Care) International. Refinement principles for animal models and experimental procedures were employed to maximize animal welfare and to optimize the experimental outcome of animal experiments. The research is aligned with the principles of the 3R’s: Replacement, Reduction, and Refinement. The University of Copenhagen is registered with the National Institutes of Health, the Animal Welfare Assurance Number is F16-00203 (A5846-01).

### Human islets

Human pancreatic islets of Langerhans isolated by collagenase digestion were received from the European Consortium for Islet Transplantation (ECIT), hand-picked, and maintained in RPMI-1640 medium w/o glucose supplemented with 10% FBS, 1% P/S and 5.6 mM D-glucose (Life Technologies). For experiments, a mixture of TNF-α, IL-1β and IFN-γ was generally used to exploit the synergy between these three cytokines for induction of more robust responses, since human islets are more resistant to cytokines than mouse and rat islets [1]. Human pancreatic islets were used for quantitative real time PCR (qRT-PCR) of miR-146a-5p.

Islets from nine non-diabetic necrodonors, five from male donors and three from female donors (age 8–57 years), were exposed for 48 h to a mixture of TNF-α (5000 U/ml, Peprotech), IFN-γ (750 U/ml, BD Pharmingen) and IL-1β (75 U/ml, Sigma).

In addition, islets were obtained from three different non-diabetic necrodonors, one 64 year-old male and two 22 and 52 year-old females, respectively. Threehundred and fifty islets per condition were transferred to medium at receipt as described above but with 2% human serum instead of FBS. After a 24 h pre-incubation, the islets were treated with or without 500 nM Givinostat (SelleckChem) 1 h prior to cytokine exposure to a mixture of 300 pg/ml recombinant mIL-1β (R&D systems), 10 ng/ml recombinant hIFN-γ (BD Biosciences) and 10 ng/ml recombinant hTNF-α (Peprotech) for 24 h.

### Microarray analysis

INS1 cells were seeded in 6-well plates, incubated for 48 h, and left untreated, treated with 125 nM Givinostat (Giv) 1 h prior to the addition of 150 pg/ml IL-1β and 0.1 ng/ml IFN-γ (Cyt) or no cytokines (ctrl) for 6 h or 24 h. miR-enriched total RNA was purified with the miRCURY^™^ RNA Isolation Cell and Plant Kit (Exiqon). The samples for microarray screening were divided into 8 groups (6h_Ctrl, 6h_Cyt, 6h_Giv_Ctrl, 6h_Giv_Cyt, 24h_Ctrl, 24h_Cyt, 24h_Giv_Ctrl, and 24h_Giv_Cyt) with each group containing three replicates. A total of 24 samples where hybridized to SurePrint G3 8x15k Rat miRNA Agilent microarray (G4473C). The microarray platform contained 12843 probes including 434 negative controls, 281 positive controls, and 12128 miRs. A total of 758 unique miRs were present on the array and each miR represented by 16 probes.

The microarray data analyses were performed using Bioconductor packages in R programming language [28]. The microarray quality control was assessed using the R Bioconductor package arrayQualityMetrics [29]. Raw data import and normalization together with differential expression analysis was performed using the R Bioconductor package limma [30]. The signal intensity of each probe on each array was quantified by taking the median foreground signal with no background correction. Subsequently all arrays were filtered to remove control probes and were normalized using the quantile normalization method which sets intensities to have the same empirical distribution across arrays. The differential expression analysis was performed in limma where a moderated t-statistic is computed for each contrast for each probe. Limma performs the multiple probe-to-miR mapping using a pooled correlation method, where correlation among replicated miR probes is taken into account in the model. Differentially expressed miRs were identified in the following 12 contrasts: 6h_Cyt vs 6h_Ctrl, 6h_Giv_Cyt vs 6h_Cyt, 6h_Giv_Cyt vs 6h_Giv_Ctrl, 6h_Giv_Ctrl vs 6h_Ctrl, 24h_Cyt vs 24h_Ctrl, 24h_Giv_Cyt vs 24h_Cyt, 24h_Giv_Cyt vs 24h_Giv_Ctrl, 24h_Giv_Ctrl vs 24h_Ctrl, 24h_Cyt vs 6h_Cyt, 24h_Giv_Cyt vs 6h_Giv_Cyt, 24h_Ctrl vs 6h_Ctrl and 24h_Giv_Ctrl vs 6h_Giv_Ctrl. p-values were adjusted for multiple correction using Benjamini and Hochberg’s method. A cutoff of abs-log2(FC) >0.1375 and adjusted p-value <0.05 were used to identify differentially expressed miRs in each contrast. The cutoff for log2(FC) corresponds to a fold change of 1.15, and since the dynamic range of microarray is not as high as for qRT-PCR, a fold change of 1.15 on microarray normally corresponds to a much higher change in qRT-PCR validated miR levels. Further, for miR qRT-PCR data, a fold change of 1.2 is generally used as cutoff instead of the 2-fold cutoff for mRNAs, since it is believed that small differences in miR levels are sufficient for the fine-tuning of the transcriptional system executed by miRs. Various studies have shown miRs with a fold change ~1.2 to be biologically relevant [31, 32].

### RNA purification and qRT-PCR evaluation of miRs

INS1 cells were seeded in 6-well plates, incubated for 48 h, and left untreated, treated with 1 μM of the KDACi CI994 or 125 nM Givinostat (Giv) 1 h prior to the addition of 150 pg/ml IL-1β and 0.1 ng/ml IFN-γ (Cyt) or no cytokines for 6 h, 18 h or 24 h. miR-enriched total RNA was purified with the miRCURY^™^ RNA Isolation Cell and Plant Kit (Exiqon). SYBR Green-based qRT-PCR quantification of specific miRs was performed using the Universal cDNA Synthesis Kit II and ExiLENT SYBR^®^ Green master mix (Exiqon) according to the manufacturer’s protocols with the following modifications: 20 ng/μl total RNA in 10 μl cDNA reactions, and 40X cDNA dilutions in 10 μl qPCR reactions were used. Primer efficiencies were all in the range of 90–108%.

### RNA purification and qRT-PCR for miR-146a-5p, *TRAF6* and *IRAK1*

RNA was extracted using TRIZOL reagent (Invitrogen), as described by the manufacturer. RNA quality and concentration was measured on a NanoDrop 1000 (Thermo Scientific). cDNA for qRT-PCR was prepared using the TaqMan^®^ Reverse Transcription kit (Applied Biosystems), according to the manufacturer’s protocol. cDNA for mature miR expression was prepared using TaqMan^®^ microRNA Reverse Transcription kit (Applied Biosystems) according to a protocol optimized for multiplex reactions with up to four miR primer sets. CT level did not differ when comparing singleplex and multiplex reactions. The cDNA synthesis was performed on an ABI thermal cycler 3100 (Applied Biosystems).

Gene and miR expressions were analyzed by qRT-PCR using SYBR green and TaqMan assays (Applied Biosystems), as described by the manufacturer. Samples were analyzed in duplicates on an ABI 7900 HT platform (Applied Biosystems). Data was examined using the SDS 2.1 software (Applied Biosystems) and evaluated by the 2^**-ΔΔ*Ct***^ method described in [33]. The internal control gene *PPIA* was used for normalizing *TRAF6* and *IRAK1* expression. Let-7c was tested thoroughly for stable expression during cytokine treatment and used as internal control for normalizing miR-146a-5p expression in samples from human, rat and mouse. Alternatively, the geometric mean of miR-103a and miR-423 was used, as indicated in the figure legends.

### Western blotting

Protein concentrations were measured by the Bradford method, according to the manufacturer’s instructions (BioRad). Samples containing equal amounts of protein were prepared with loading buffer and boiled before loading on a Nupage Novex gel (Invitrogen). The gel was placed in an X-cell Surelock system (Invitrogen), and proteins were separated. The proteins were then transferred to a nitrocellulose membrane (Invitrogen), which was blocked in skim milk or BSA. Primary antibodies were preabsorbed with milk or BSA. Visualization involved HRP-linked secondary antibodies. Proteins of interest were detected with Lumi-GLO (Cell Signaling) and visualized using a LAS3000 imaging system (Fuji Film). Antibodies were rabbit anti-TRAF6 (Santa Cruz, #sc-7221), rabbit anti-inducible nitric oxide synthase (iNOS) (BD Biosciences, #610332), rabbit anti-IRAK1 (Abcam, #AB238), rabbit anti-phospho-c-Jun N-terminal kinase 1/2 (JNK1/2) (Cell Signaling, #9251), rabbit anti-phospho-p38 (Cell Signaling, #9211), rabbit anti-phospho-extracellular signal-regulated kinase 1/2 (ERK1/2) (Cell Signaling, #9101), and mouse anti-β-actin (Abcam, #AB6276-100). Anti-rabbit IgG (Cell Signaling, #7074) and anti-mouse IgG (Abcam, #Ab6789) were used as secondary antibodies.

### Luciferase promoter and 3’UTR constructs

Vectors with the luciferase gene and the native 3’untranslated region (3’UTR) construct of TRAF6 or IRAK1, described in [34], were purchased from AddGene (www.Addgene.org). The native iNOS promoter construct was described in [35], and the NF-κB promoter construct (pNF-κB) containing a repetitive κB motive was purchased from Stratagene. The miR-146a-5p promoter construct, described in [34], was also ordered from AddGene. The luciferase activity was measured by a dual luciferase reporter assay system (Promega), according to the manufacturer’s protocol. The luciferase activity was normalized to a co-transfected vector (pRL-TK) containing a HSV-TK promoter driven Renilla construct (Promega). Readings were conducted on a luminometer from Berthold Technologies.

### Transfection with miR

INS1 cells were transfected with miR-146a-5p miRIDIAN mimics (#C-300630-03, Thermo Scientific) or a negative control miR mimic (#CN-001000-01, Thermo Scientific) using DharmaFECT 1 (Thermo Scientific) as transfection reagent. All transfections had a final oligo concentration of 30 nmol/l. Cell medium was changed at the day of transfection to medium without antibiotics. In two separate tubes, OptiMEM (Invitrogen) was mixed with DharmaFECT-1 reagent and with synthetic oligos, and the two tubes were incubated for 5 min at room temperature. The contents of the two tubes were mixed 1:1 and incubated at room temperature for 20 min. The transfection solution was then diluted 1:5 with RPMI-1640 (Invitrogen) and added to the cells. The transfection medium was removed after overnight incubation at 37°C in a humidified atmosphere of 5% CO_2_. The cells were then incubated for at least another 24 h. Transfection efficiency was estimated to ~50% by co-transfection with a GFP expressing plasmid pMAX (Lonza). Furthermore, the expression level of the transfected miR mimic was examined as a transfection control. For evaluation of miR overexpression in combination with cytokine stimulation, cells were pre-treated with miR mimics for 48 h and then exposed to IL-1β (160 pg/ml) for 30 min or 6 h.

### Apoptosis

INS1 cells were left untreated, treated with 1 μM CI994 or 125 nM Givinostat 1 h prior to addition of 0.1 ng/mL IFN-γ plus 37–150 pg/mL IL-1β or no cytokines for 24 h. Apoptosis was measured using the Cell Death Detection ELISA (Roche) according to the manufacturer’s protocol.

### Nitric oxide (NO) measurements

The INS1 cell production of NO was measured as accumulated nitrite in the culture medium using the Griess reagent, since NO is rapidly converted to nitrite. Supernatants from stimulated cells were mixed with equal volumes of cell culture medium and Griess reagent (0.1% [wt/vol.] naphthylethene diamine hydrochloride; Sigma) in H_2_O, and 1% (wt/vol.) sulphanilamide (Bie & Berntsen) in 5% (vol./vol.) H_3_PO_4_ (Merck). Absorbance was measured at 550 nm, and accumulated nitrite was calculated from a NaNO_2_ standard curve [19].

### miR target prediction and bioinformatics analysis

The CyTargetLinker plugin v 3.01 [36] within Cytoscape [37] was used for identifying molecular targets for each miR and to create miR-target interaction (MTI) networks. Regulatory interaction networks (RegIN) were created within CyTargetLinker in Cytoscape using Homo sapiens MTIs from one experimentally validated miR-target resource miRTarBase v6.1 [38] which includes 410,602 MTIs, and one predicted miR-target resource TargetScan v6.2 [39], which includes 511,040 MTIs. Only those targets were kept that were supported by both of these resources and visualized in networks.

Functional annotation clustering module in DAVID (the Database for Annotation, Visualization, and Integrated Discovery) [40] was used to cluster similar gene ontology (GO) annotation terms associated with the identified miR-targets into functionally annotated clusters. GO-Fat biological process (BP) categories were used for the analysis. An EASE score (modified Fisher’s exact t-test) cutoff of <0.01 and highest stringency classification was applied. A group enrichment score in DAVID is calculated by taking the geometric mean (in -log scale) of each member annotation term’s p-values in a corresponding annotation cluster. The enriched clusters with group enrichment scores > 1.3 were selected.

### Statistical analysis

The microarray analyses were done in R programming language using the Bioconductor packages arrayQualityMetrics and limma, as described above. All other statistical analyses were carried out in GraphPad Prism software v7.0 or Microsoft Excel. Two-tailed Student’s t-test was used to analyze significant differences between two groups, unless otherwise stated. All one-way or two-way ANOVA analyses were corrected for multiple testing using Bonferroni test. p-values below 0.05 were considered statistically significant.

## Results

### KDAC inhibition regulates cytokine-induced miR expression

To investigate whether the mechanisms behind KDACi-mediated protection against cytokine-induced apoptosis involve transcriptional regulation of miRs, we performed a miR array profiling of 758 rat miRs (miRbase version 21.0). With the purpose of selecting the experimental setup for the miR array, insulin-producing INS1 cells were cultured with or without the KDACi CI994, an inhibitor selective for HDAC1, 2, and 3, or the broad-spectrum KDACi Givinostat, prior to exposure to different concentrations of the pro-inflammatory cytokine IL-1β (37–150 pg/ml) together with IFN-γ (0.1 ng/ml) for 6 h or 24 h. As seen in S1 Fig, both CI994 and Givinostat reduced cytokine-induced cell death and NO production, confirming previous results from our group and others [16, 19]. Given the higher efficacy of Givinostat in lowering NO production, this KDACi was chosen for the miR array study. INS1 cells were exposed to Givinostat or no treatment for 1 h and then to media with or without 150 pg/ml IL-1β and 0.1 ng/ml IFN-γ for 6 h or 24 h. RNA extracted from control (Ctrl), cytokine (Cyt), Givinostat+cytokines (Giv_Cyt), and Givinostat-cytokines (Giv_Ctrl) samples at 6 h and 24 h were used in triplicates for miR array (Table 1). Table 1 shows the numbers of significantly regulated miRs following a cut-off of adjusted p<0.05 and log2(FC) > 0.1375, corresponding to a fold change of 1.15, when analyzed for all 12 pairwise comparisons. The raw data from the miR array are deposited at GEO database (GSE117451). As expected, the addition of cytokines led to the largest change in miR expression profile, but only after 24 h. Remarkably, the addition of Givinostat also changed the expression of a substantial numbers of miRs.

**Table 1:** Summary of miR microarray results.

Comparison	Biological significance of comparison	# of miRs significantly regulated in given comparisons
6 h_Cyt vs 6 h_Ctrl	Regulated by cytokines at 6 h	2
6 h_Giv_Cyt vs 6 h_Cyt	Regulated by Giv.in presence of cytokines at 6 h	21
6 h_Giv_Cyt vs 6 h_Giv_Ctrl	Regulated by cytokines in presence of Giv. at 6 h	-
6 h.Giv.Ctl vs 6 h_Ctrl	Regulated by Giv.in absence of cytokines at 6 h	5
24 h_Cyt vs 24 h_Ctrl	Regulated by cytokines at 24 h	48
24 h_Giv_Cyt vs 24 h_Cyt	Regulated by Giv. in presence of cytokines at 24 h	24
24 h_Giv_Cyt vs 24 h_Giv_Ctrl	Regulated by cytokines in presence of Giv. at 24 h	43
24 h_Giv_Ctrl vs 24 h_Ctrl	Regulated by Giv.in absence of cytokines at 24 h	3
24 h_Cyt vs 6 h_Cyt	Difference in effects of cytokine exposure for 24 h vs. 6 h	18
24 h_Giv_Cyt vs 6 h_Giv_Cyt	Difference in effects of Giv. exposure for 24 h vs. 6 h in the presence of cytokines	14
24 h_Ctrl vs 6 h_Ctrl	Difference between 24 h vs. 6 h in absence of cytokines and Giv.	5
24 h_Giv_Ctrl vs 6 h_Giv_Ctrl	Difference in effects of Giv. exposure for 24 h vs. 6 h in the absence of cytokines	2

The numbers denote significantly regulated miRs following a cut-off of adjusted p<0.05 and log2(FC) >0.1375, corresponding to a fold change of 1.15. Shadings denote pairwise comparisons of interest regarding the scientific hypothesis.

We next analyzed which miRs were significantly regulated both by cytokines and by Givinostat in the presence of cytokines. These were divided into four groups based on their regulation patterns, as shown in Table 2.

**Table 2:** miRs regulated by cytokines and Givinostat.

			**Log2FC**		**Adjusted p-value**	
**Group**	**MicroRNA**	**Human ortholog**	**24 h_Cyt vs Ctrl**	**24 h_Giv_Cyt vs Cyt**	**24 h_Cyt vs Ctrl**	**24 h_Giv_Cyt vs Cyt**
A: Cyt↓, Giv↑, 24 h	rno-miR-7a-2-3p	hsa-miR-7-2-3p	-0.22	0.36	8.90E-03	6.71E-06
	rno-miR-466b-2-3p	hsa-miR-466	-0.26	0.30	4.96E-02	3.29E-02
	rno-miR-652-5p	hsa-miR-652-5p	-0.56	0.32	1.20E-27	2.66E-09
B: Cyt↑, Giv↓, 24 h	rno-miR-29c-3p	hsa-miR-29c-3p	0.71	-0.20	5.75E-35	2.61E-03
	rno-miR-96-5p	hsa-miR-96-5p	0.70	-0.24	3.19E-36	6.24E-05
	rno-miR-101a-3p	hsa-miR-101-3p	0.60	-0.30	2.90E-33	7.85E-09
	rno-miR-140-5p	hsa-miR-140-5p	0.17	-0.13	5.05E-05	8.10E-03
	rno-miR-340-5p	hsa-miR-340-5p	0.39	-0.22	1.91E-24	5.44E-08
	rno-miR-384-5p	hsa-miR-384	0.38	-0.30	1.44E-09	2.48E-05
	rno-miR-455-5p	hsa-miR-455-5p	0.19	-0.15	6.91E-08	6.24E-05
	rno-miR-3584-5p	*No human ortholog*	0.38	-0.14	1.46E-32	2.68E-05
C: Cyt↑, Giv↑, 24 h	rno-miR-146a-5p	hsa-miR-146a-5p	0.59	0.16	5.17E-32	5.17E-32
**Group**	**MicroRNA**	**Human ortholog**	**6 h_Cyt vs Ctrl**	**6 h_Giv_Cyt vs Cyt**	**6 h_Cyt vs Ctrl**	**6 h_Giv_Cyt vs Cyt**
D: Cyt↑, Giv↓, 6 h	rno-miR-146b-5p	hsa-miR-146b-5p	0.44	-0.42	5.70E-07	3.02E-06

miRs fulfilling the requirements to be regulated both by cytokines and by Givinostat in the presence of cytokines, sorted into groups.

To identify the potential targets of these groups of miRs we used CyTargetLinker plugin within Cytoscape. miR-target interaction networks were created for the four selected groups of miRs as listed in Table 2 (S2 Fig). From group A with three miRs, miR-7a-2-3p and miR-652-5p did not have targets from the two target prediction resources used for this analysis (Fig A in S2 Fig). In group B with 8 miRs, miR-340-5p did not have any targets (Fig B in S2 Fig). Interestingly, the shared targets of miR-29c-3p, miR-96-5p, and miR-101-3p of group B were found to be related to multiple cellular functions and interconnected in a larger network (Fig B in S2 Fig). miR-466 and miR-455-5p from Group A and B, respectively, formed discrete non-interconnected target networks (Figs A and B in S2 Fig). Of note, miR-146a-5p and miR-146b-5p from group C and D formed discrete overlapping but not identical target networks (Fig C in S2 Fig).

We next performed functional annotation clustering analysis based on biological process (BP) gene ontology (GO) terms in DAVID. We searched for functional clusters of genes within the target genes of the above mentioned four groups based on enriched BP terms. For group A miR-targets, no enriched clusters were found. For group B miR-targets, seven significantly enriched clusters were found. The top enriched clusters for group B miR-targets were associated with negative regulation of transcription, regulation of apoptosis and cell-death processes (S1 Table). In case of group C and D, three enriched clusters were found which represented NF-κB-inducing kinase activity, regulation of phosphorylation and regulation of cell development processes (S1 Table). These results highlight the involvement of miR-targets from group B, C and D in various important cellular processes.

### qRT-PCR validation of specific miRs

**Fig 1:**
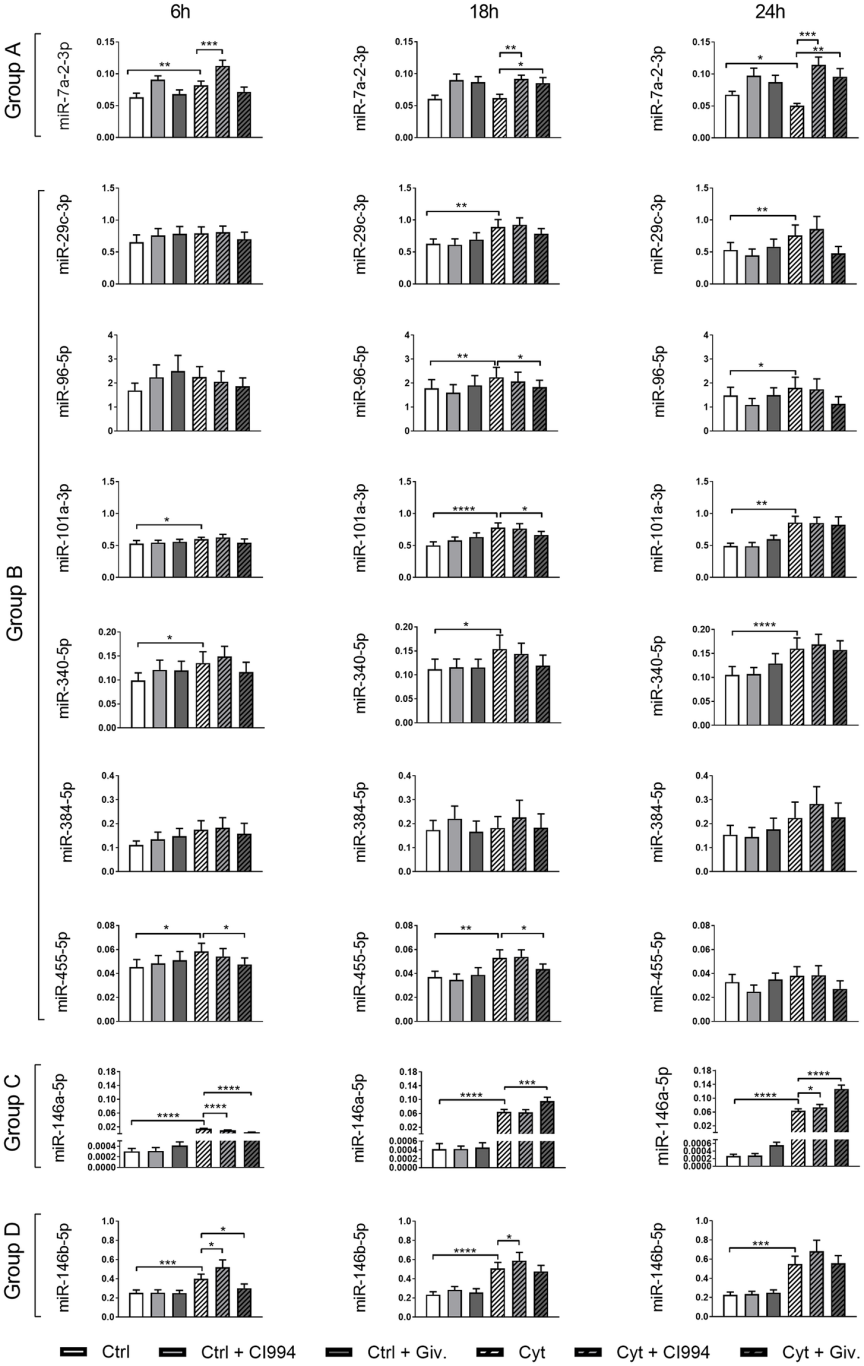
Expression of selected miRs in INS1 cells. INS1 cells were left untreated, treated with 1 μM of the KDACi CI994 or 125 nM Givinostat (Giv) 1 h prior to the addition of 150 pg/ml IL-1β and 0.1 ng/ml IFN-γ (Cyt) or no cytokines for 6 h, 18 h or 24 h. MiR-enriched total RNA was purified, and the expression of specific miRs was measured with qRT-PCR and normalized to the geometric mean of the housekeeping miRs miR-103a and miR-423. Data are presented as mean ± SEM (n = 9–10) of 2^-ΔCT^. Statistical significance of a paired t-test is shown in the graphs (*p<0.05, **p<0.01, ***p<0.001 and ****p<0.0001). Group A: miRs were Cyt↓, Giv↑ at 24 h. Group 2: miRs were Cyt↑, Giv↓ at 24 h. Group C: miR was Cyt↑, Giv↑ at 24 h. Group D: miR was Cyt↑, Giv↓ at 6 h.

Out of the thirteen miRs regulated by both cytokines and Givinostat, nine representative miRs were evaluated by qRT-PCR on INS1 cells treated similarly to the array samples but using both CI994 and Givinostat and 6 h, 18 h, and 24 h exposure to cytokines (Fig 1). For group A (Table 2), miR-7a-2-3p was confirmed to be downregulated after 24 h of cytokine exposure, which was prevented by Givinostat (Fig 1). For group B, upregulation after 24 h and also 18 h of cytokine exposure was confirmed for miR-29c-3p, miR-96-5p, miR-101a-3p, and miR-340-5p. Givinostat did not significantly change their expression after 24 h of cytokine exposure. However, Givinostat did significantly reduce the 18 h cytokine-induced expression of miR-96-5p, miR-101a-3p, and also miR-455-5p, the latter also after 6 h of exposure (Fig 1). For group D, cytokines persistently upregulated the expression of miR-146b-5p and the cytokine-induced upregulation was prevented by Givinostat after 6 h of exposure, confirming the array data (Fig 1).

The most remarkable miR expression pattern was that of the related miR-146a-5p in group C, which was highly upregulated at 6 h, 18 h and 24 h of exposure to cytokines (Fig 1). Moreover, ANOVA testing demonstrated significance between KDACi treatments and time of exposure, with interactions between treatments and time of exposure (S2 Table). Confirming the array data, Givinostat enhanced the cytokine-induced expression of miR-146a-5p at both 18 h and 24 h with 48% and 100%, respectively (Fig 1). In contrast, at 6 h, Givinostat reduced the cytokine-induced expression of miR-146a-5p. CI994 had a similar but smaller effect at 6 h and 24 h (Fig 1), reflecting the fact that this inhibitor targets only three KDAC enzymes, whereas Givinostat targets all non-sirtuin KDACs.

Our results demonstrate that a number of miRs are subject to KDAC-dependent expressional changes following cytokine treatment in INS1 cells. As miR-146a-5p was the most significantly regulated miR by both cytokines and Givinostat we focused on this miR in the following experiments.

### KDACi reduces cytokine-mediated miR-146a-5p induction in human islets

**Fig 2:**
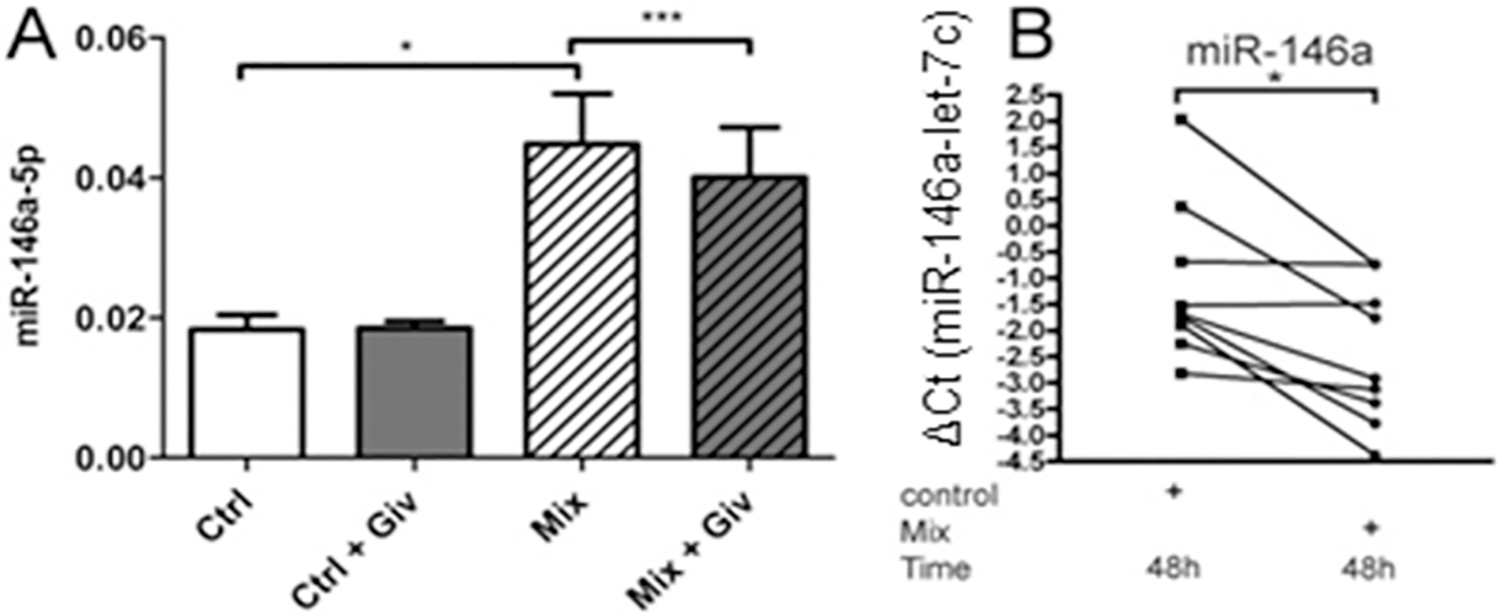
KDACi reduces cytokine-induced expression of miR-146a-5p in human islets. (A) Human islets from three individual necro-donors were treated with or without 500 nM Givinostat (Giv) 1 h prior to addition of 300 pg/ml IL-1β, 10 ng/ml IFN-γ, and 10 ng/ml TNF-α (Mix) or no cytokines (Ctrl) for 24 h. miR-146a-5p expression was measured with qRT-PCR and normalized to the geometric mean of the housekeeping miRs miR-103a and miR-423. Data are presented as mean ± SEM of 2^-ΔCT^. *p<0.05 (95% confidence interval = -0.04836 to -0.004574), ***p<0.001 (95% confidence interval = 0.004108 to 0.005359). (B) Human islets from nine individual necro-donors were left untreated (control) or treated for 48 h with a mixture of 5000 U/ml TNF-α, 750 U/ml IFN-γ, and 75 U/ml IL-1β (Mix). miR-146a-5p expression was analyzed by qRT-PCR and presented as ΔCT values after normalizing to let-7c. *p<0.05.

We next wanted to confirm cytokine-mediated upregulation of miR-146a-5p in human pancreatic islets of Langerhans. qRT-PCR analysis showed that a mixture of cytokines significantly upregulated miR-146a-5p expression in isolated human islets after 24 h of treatment (Fig 2A) as well as after 48 h of treatment (Fig 2B). In contrast to the potentiating effect of Givinostat in INS1 cells after 24 h of cytokine exposure, Givinostat reduced cytokine-induced miR-146a-5p expression in human islets (Fig 2A). However, the results confirm that cytokine-induced miR-146a-5p expression is subject to KDAC-dependent regulation in both rat insulin-secreting INS1 cells and in human islets. Based on this, we chose to further investigate the underlying mechanisms and functions of the cytokine-induced expression of miR-146a-5p.

### miR-146a-5p expression is mainly induced by IL-1β

**Fig 3:**
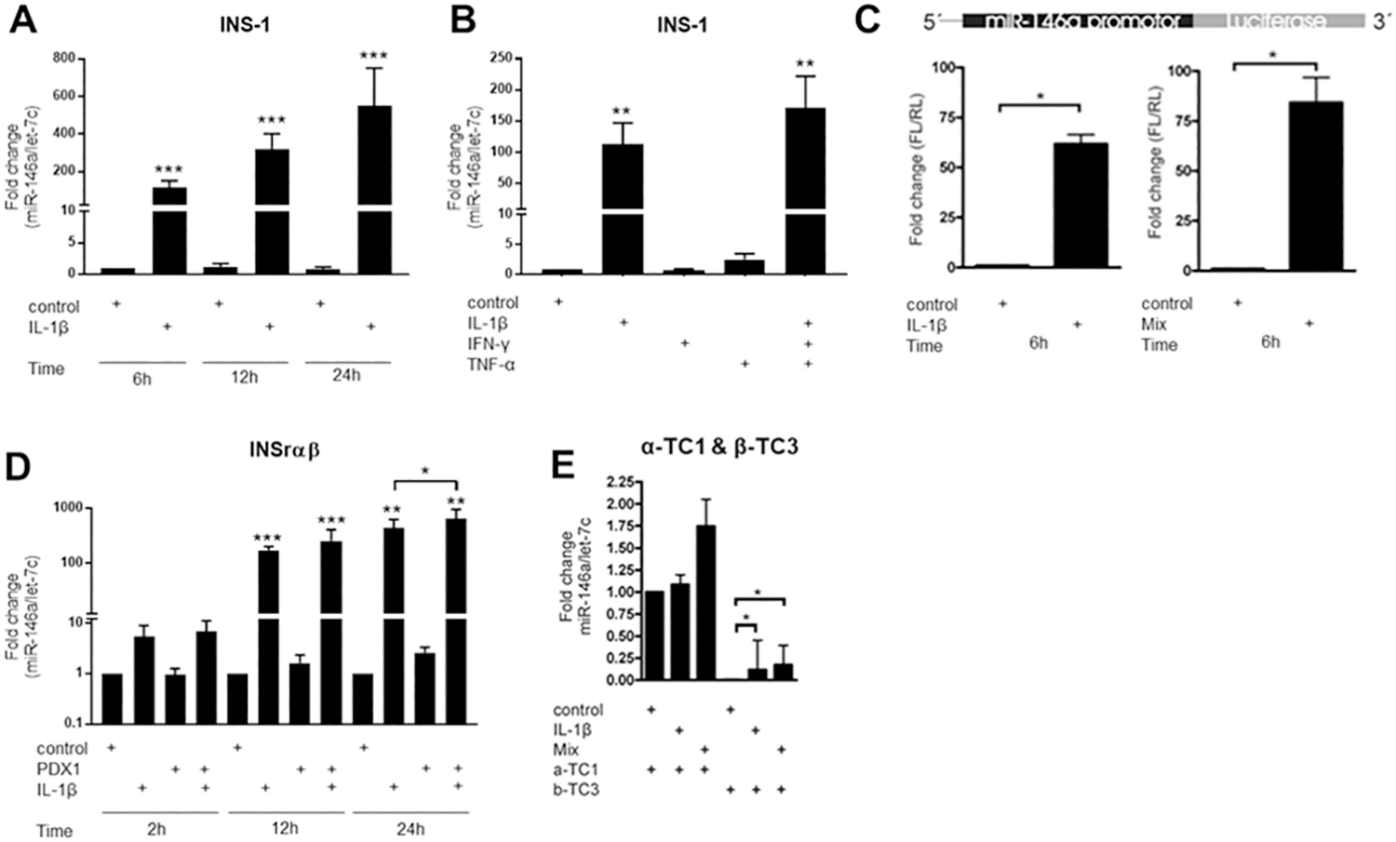
miR-146a-5p expression in α- and β-cell lines. (A) INS1 cells were exposed to IL-1β (160 pg/ml) for 6 h, 12 h and 24 h. The expression of miR-146a-5p normalized to the internal control let-7c was quantified with qRT-PCR. Mean ± SEM (n = 5). (B) INS1 cells were exposed to IL-1β (160 pg/ml), IFN-γ (5ng/ml) or TNF-α (10 ng/ml) or a mixture of all three cytokines for 8 h. The expression of miR-146a-5p normalized to the internal control let-7c was quantified with qRT-PCR. Mean ± SEM (n = 3). (C) The Luciferase promoter assay was conducted in INS1 cells exposed to IL-1β (160 pg/ml) for 6 h or IL-1β (160 pg/ml) and IFN-γ (5 ng/ml) for 6 h. The cells were pre-treated with transient transfection mix containing both miR-146a-5p promoter construct and a Renilla construct. Mean ± SEM (n = 4). (D) miR-146a-5p expression in the INSrαβ cell line with or without induced Pdx-1 by addition of doxycycline for 24 h prior to cytokine exposure to IL-1β (150 pg/ml) for 2 h, 12 h, or 24 h. The expression of miR-146a-5p and let-7c was quantified using qRT-PCR. Mean ± SEM (n = 4–5). (E) α-TC1 and β-TC3 cells were exposed for 24 h to IL-1β (160 pg/ml) or IL-1β (160 pg/ml) and IFN-γ (5 ng/ml). The expressions of both miR-146a-5p and the internal control let-7c were quantified by qRT-PCR. Mean ± SEM (n = 6–8). *p<0.05; ** p<0.01; *** p<0.001.

Next, we wished to detail further the induction of miR-146a-5p by IL-1β alone versus a mixture of cytokines. The expression of miR-146a-5p in INS1 cells was markedly and significantly upregulated upon stimulation with IL-1β alone for 6 h, 12 h or 24 h, respectively (Fig 3A). Neither TNF-α nor IFN-γ alone had a significant effect on miR-146a-5p expression at 8 h exposure, whereas a mixture of IL-1β, IFN-γ and TNF-α for 8 h resulted in induction of miR-146a-5p at levels only slightly higher than IL-1β alone (Fig 3B). We also examined the expression of miR-146a-5p in rat islets exposed to cytokines. miR-146a-5p was induced by IL-1β alone and more profoundly so by a combination of IL-1β, IFN-γ and TNF-α in a time-dependent manner with peak induction at the latest time point of 24 h (S3 Fig).

Using constructs containing the native human miR-146a-5p promoter driving Firefly luciferase expression as previously described [34], we investigated whether IL-1β alone or in combination with IFN-γ was able to induce expression of the luciferase gene. We found that 6 h of cytokine stimulation significantly induced the Firefly luciferase expression through the miR-146a-5p promoter, both by IL-1β alone and slightly more by a mixture of IL-1β, IFN-γ and TNF-α (Fig 3C).

### IL-1β-induced expression of miR-146a-5p is differentiation dependent

Because β-cell sensitivity to cytokines is acquired during β-cell maturation [41], we wished to examine whether cytokine-mediated induction of miR-146a-5p is also an acquired trait of the β-cell phenotype. For these studies we used the INSrαβ cells, which in response to Pdx-1 induction progress from an immature to a mature β-cell phenotype [26, 42]. Fig 3D shows the effect of Pdx-1 induction and/or IL-1β exposure on the expression of miR-146a-5p. The expression of miR-146a-5p was highly upregulated in response to stimulation with IL-1β both alone (12 h: 170 fold; 24 h: 440 fold) and together with Pdx1 induction (12 h: 250 fold; 24 h: 660 fold). The expression of miR-146a-5p was not affected by Pdx-1 induction alone, but after 24 h Pdx-1 induction led to an increase in IL-1β-induced miR-146a-5p expression, suggesting that this regulation is differentiation dependent.

Next, we compared the expression of miR-146a-5p in the mouse β- and α-cell models β-TC3 and α-TC1 cells to validate the findings in the maturation system. In β-TC3 cells, the expression of miR-146a-5p was significantly increased in response to IL-1β alone or in combination with IFN-γ after 24 h of exposure (Fig 3E). The α-TC1 cells showed no significant changes in miR-146a-5p expression upon cytokine treatment. Interestingly, the basal expression of miR-146a-5p was much higher in α-TC1 versus β-TC3 cells. There were no differences between the two cell lines in the expression of the internal controls Let-7c and *Ppia*. Taken together, these results suggest that miR-146a-5p is induced by cytokines preferentially in β-cells.

### miR-146a-5p overexpression inhibits NF-κB signaling, blocks iNOS expression, and targets TRAF6 and IRAK1

**Fig 4:**
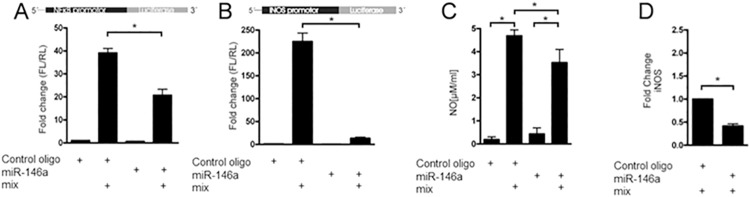
miR-146a-5p overexpression inhibits NF-κB signaling and blocks iNOS expression. (A) The Luciferase promoter assay was performed in INS1 cells with a NF-κB promoter construct driving Firefly luciferase. The cells were pre-treated with transient transfection mix containing both miR-146a-5p promoter construct and a Renilla construct as internal control. Additionally, the INS1 cells were transiently transfected with a control oligo or a synthetic miR-146a-5p oligo, and exposed to media with or without IL-1β (160 pg/ml) for 6 h. The relative fold change in ratio between Firefly/Renilla luciferase is plotted. Mean ± SEM (n = 4). (B) iNOS activity was measured by luciferase assay as described above. Mean ± SEM (n = 4). (C) NO in medium from INS1 cells transfected with either a control oligo or a synthetic miR-146a-5p 24 h prior to exposure to IL-1β (160 pg/ml) and IFN-γ (5 ng/ml). Mean ± SEM (n = 4). (D) iNOS protein expression was analyzed by Western blotting from INS1 cells transfected with either a control oligo or a synthetic miR-146a-5p 24 h prior to exposure to IL-1β (160 pg/ml) and IFN-γ (5 ng/ml). Mean ± SEM (n = 4). *p<0.05.

The effects of miR-146a-5p on cytokine-stimulated NF-κB and iNOS promoter activities were investigated by the use of promoter-luciferase constructs in INS1 cells (Fig 4). Transfection with miR-146a-5p significantly reduced IL-1β-stimulated NF-κB promoter activity by ~50%, as compared to transfection with a control miR oligo (Fig 4A), indicating that miR-146a-5p inhibits this signaling pathway. Furthermore, miR-146a-5p was able to completely block IL-1β-induced iNOS promoter activity (Fig 4B), and transfection with miR-146a-5p significantly decreased cytokine-induced NO production as compared to transfection with a control miR oligo (Fig 4C). Consistent with these observations, miR-146a-5p transfection caused a ~50% reduction in IL-1β-induced iNOS protein expression (Fig 4D and Fig A in S4 Fig).

TRAF6 and IRAK1 are known validated targets of miR-146a-5p [34, 43], and we wished to confirm this in INS1 cells. Transfection with miR-146a-5p mimics reduced TRAF6 and IRAK1 protein expression in the presence and absence of IL-1β, and co-transfection with anti-miR-146a-5p restored their expression (Fig A in S4 Fig). We then investigated whether miR-146a-5p was able to target a reporter gene construct containing the luciferase gene and the native 3’UTR of TRAF6 or IRAK1 by co-transfecting each construct with miR-146a-5p mimics. Tranfection with miR-146a-5p caused a ~50% reduction in luciferase activity of both the TRAF6 and IRAK1 3’UTR reporters, whereas blocking miR-146a-5p with anti-miR-146a-5p restored and enhanced the luciferase signal (Figs B and D in S4 Fig). Next, the effect of miR-146a-5p on the mRNA expression of TRAF6 and IRAK1 was examined. Transfection with miR-146a-5p reduced the mRNA expression levels of TRAF6 and IRAK1 by ~30%, as compared to transfection with a control oligo (Figs C and E in S4 Fig). These results confirm that TRAF6 and IRAK1 are targets of miR-146a-5p in β-cells, as previously reported [34, 43].

### miR-146a-5p inhibits IL-1β-induced MAPK activation

**Fig 5:**
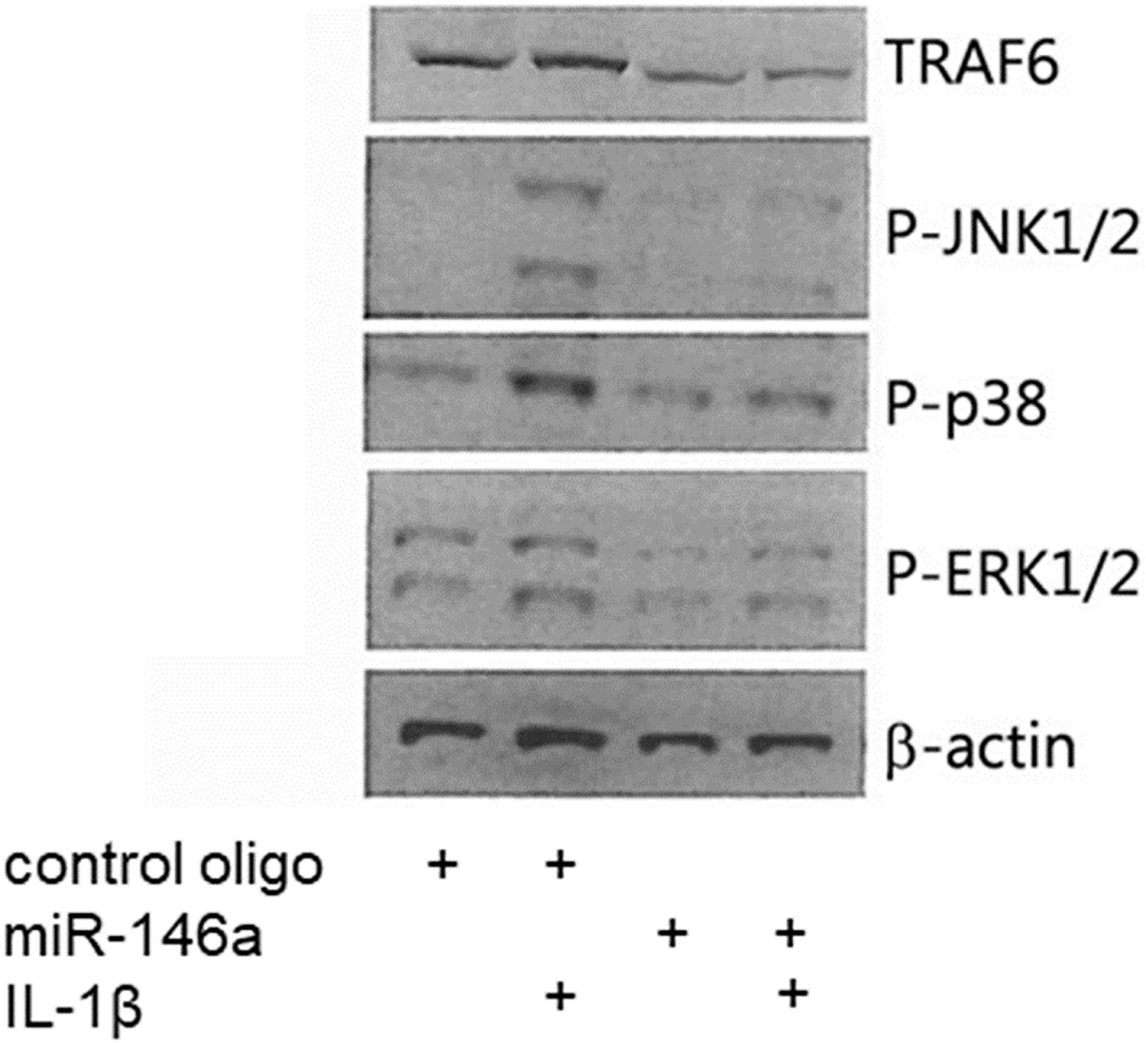
miR-146a-5p inhibits IL-1β-induced phosphorylation of JNK, p38 and ERK MAPK. Representative Western blot of TRAF6, p-JNK1/2, p-p38, p-ERK1/2, and β-actin (n = 4). INS1 cells were transiently transfected with a negative control oligo or a synthetic miR-146a-5p oligo for 48 h, and exposed to media with or without IL-1β (160 pg/ml) for 30 min.

To gain further information about the ability of miR-146a-5p to interfere with IL-1β signaling, we investigated the effects of transfection with miR-146a-5p on IL-1β-induced phosphorylations of JNK, p38, and ERK MAPKs which are key signaling components in IL-1β signal transduction. Following transfection of INS1 cells with miR-146a-5p or a negative control miR, cells were stimulated with IL-1β for 30 min, and cell lysates were analyzed by immunoblotting. The protein level of the validated miR-146a-5p target TRAF6 was greatly reduced by transfection with miR-146a-5p, confirming successful delivery of this miR (Fig 5). miR-146a-5p caused a clear reduction in IL-1β-induced phosphorylations of JNK1/2, p38 and ERK1/2 (Fig 5), suggesting that miR-146a-5p inhibits IL-1β signaling at a level proximal to MAPKs.

### miR-146a-5p expression correlates with diabetes onset in the diabetes-prone BB-DP rat

**Fig 6:**
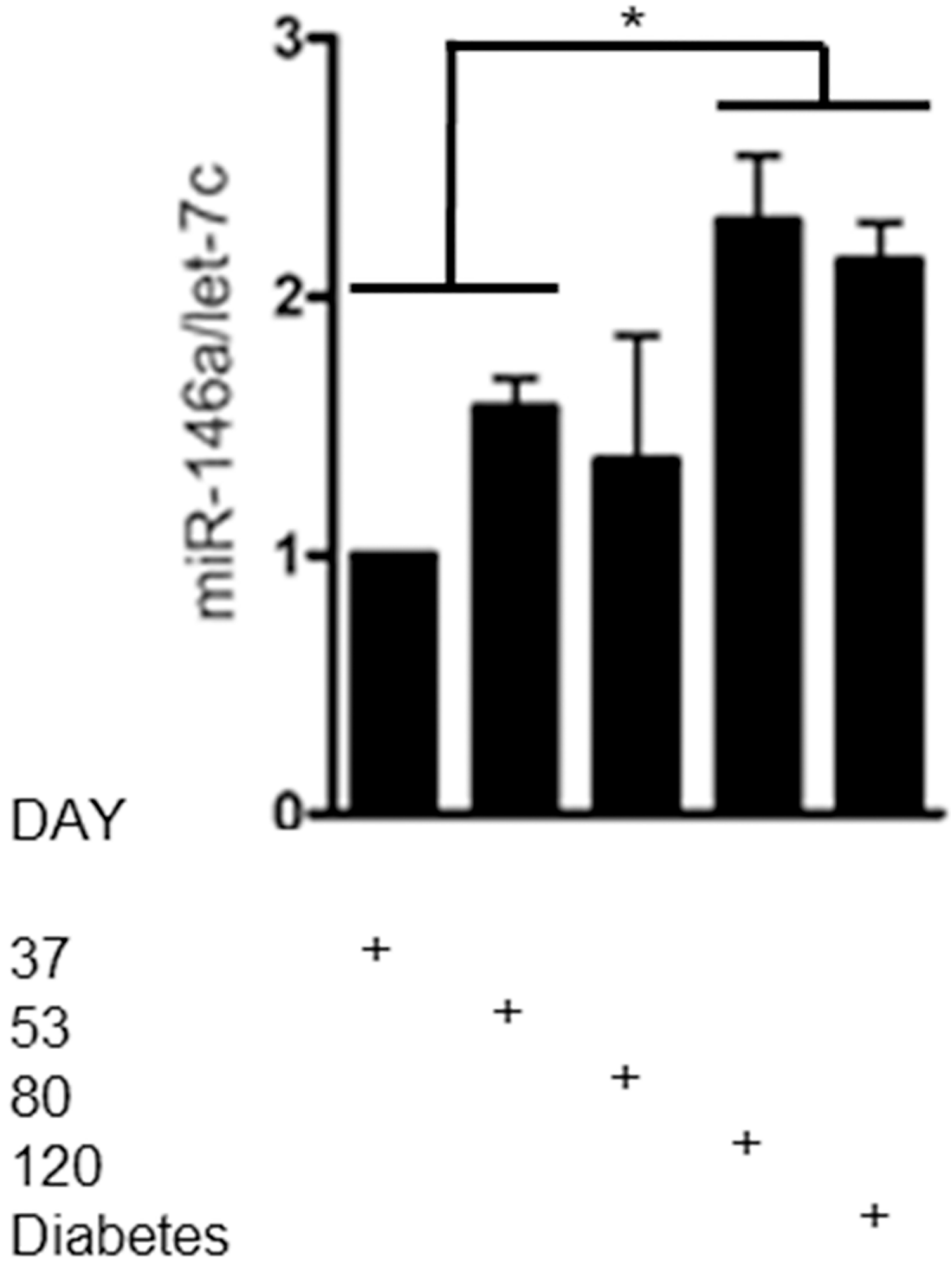
miR-146a-5p expression in the BB-DP rat T1D animal model. The expressions of miR-146a-5p and the internal control let-7c were analyzed by qRT-PCR on whole pancreas RNA from rats sacrificed at day 37, 53, 80, 120 or at diabetes onset (n = 3–6 in each group). Mean ± SD of data plotted as fold change compared to day 37. *p<0.05.

Lastly, to provide evidence for a potential role of miR-146a-5p during T1D development, its expression level was examined in the pancreas from BB-DP rats, an animal model of spontaneous autoimmune T1D, at different ages. The miR-146a-5p expression level in pancreata from 120 day old and overtly diabetic BB-DP rats showed a significant increase in miR-146a-5p expression compared with pre-diabetic rats (combined group of 37- or 53-day old rats) (Fig 6). These data show that the pancreatic expression level of miR-146a-5p gradually increased over age peaking around the onset of diabetes in BB-DP rats.

## Discussion

In this study, we pursued to identify miRs potentially involved in the protective effect of KDACi against cytokine-induced β-cell apoptosis, with the perspective of identifying miRs as novel modifiers of diabetes pathogenesis. Our miR microarray identified thirteen miRs (miR-7a-2-3p, miR-29c-3p, miR-96-5p, miR-101a-3p, miR-140-5p, miR-146a-5p, miR-146b-5p, miR-340-5p, miR-384-5p, miR-455-5p, miR-466b-2-3p, miR-652-5p, and miR-3584-5p) that were regulated by both pro-inflammatory cytokines and by the pan-KDACi Givinostat, and nine of these were examined by qRT-PCR. Being the most markedly regulated miR in the miR array and qRT-PCR analyses, we chose to focus our functional studies on miR-146a-5p.

The expression of miR-146a-5p has previously been shown to be induced by pro-inflammatory cytokines in human islets and in the mouse β-cell line MIN6 [43, 44]. Here, we have confirmed that miR-146a-5p was highly upregulated by cytokines in rat and human islets as well as in the rat β-cell line INS1, likely as an adaptive response ensuing IL-1β-induced NF-κB activation, since the miR-146a promoter contains NF-κB binding elements [34, 43, 45]. Of note, a study in human alveolar epithelial cells showed that IL-1β-induced miR-146a-5p expression is dependent on both NF-κB and JNK1/2 [46], which could also be a possible regulatory mechanism in β-cells, since IL-1β induces JNK1/2 activity in islet cells [47].

KDACi reduced the cytokine-induced expression of miR-146a-5p in INS1 cells after 6 h and in human islets after 24 h, reflecting the known action of KDACi to block inflammatory cytokine signaling by reducing NF-κB transcriptional activity [14]. In contrast, KDACi enhanced the cytokine-induced miR-146a-5p expression in INS1 cells after 18 h and 24 h of exposure. In INS1 cells cytokine signaling inducing inflammatory gene expression is rapid (<6 h) [14], and gene expression patterns determined after 24 h of cytokine exposure in INS1 cells are therefore a combination of alterations secondary to changes in the transcription of early immediate response genes and compensatory/restorative responses. In human islets, such responses would be expected to be delayed relative to those observed in INS1 cells due to the known lower sensitivity of human islets to cytokines [3] and would only be apparent in prolonged time-response experiments. The early transient reduction in miR-146a-5p expression may therefore be collateral effect of KDACi on the transcriptional activity of NF-κB, whereas a later more persistent augmentation of cytokine-induced miR-146a-5p expression is likely to be a protective response caused by histone hyperacetylation of the chromatin region harboring the miR-146a-5p gene, and directed at counteracting the adverse impact of inflammation. In support of this, previous studies have shown that KDACi increased IL-1β-induced miR-146a-5p expression in osteoarthritis fibroblast-like synoviocytes [48] and upregulated basal as well as lipopolysaccharide (LPS)-stimulated miR-146a-5p expression in macrophages isolated from mice [49].

miR-146a-5p is a known inhibitor of IL-1 signaling in many cell types at least in part by directly targeting TRAF6 and IRAK1 [34, 43, 50]. Roggli *et al*. showed that transfection with miR-146a-5p mimics reduced basal and cytokine-induced TRAF6 and IRAK1 reporter activity in MIN6 cells [43]. Here, we confirmed a reduction in the reporter activity as well as mRNA and protein levels of TRAF6 and IRAK1 in INS1 cells after transfection with miR-146a-5p mimics, supporting the evidence that TRAF6 and IRAK1 are direct targets of miR-146a-5p in β-cells. Transfection of INS1 cells with miR-146a-5p also reduced the activity of the NF-κB and iNOS promotors resulting in decreased cytokine-induced iNOS protein expression and NO synthesis. Cytokine-induced MAPK signaling was also decreased after transfection with miR-146a-5p. Our results indicate that miR-146a-5p downregulates islet inflammation and β-cell death in part by impaired NF-κB and MAPK signaling, which is in line with studies in other cell types such as lymphocytes, adipocytes, and a renal cell line [51–53].

To our knowledge, this is the first report to show islet cell selectivity in inflammatory miR-146a-5p induction. We found that cytokines only increased miR-146a-5p expression in a β-cell (β-TC3) but not in an α-cell (α-TC1) line. Furthermore, a surprisingly high basal expression level of miR-146a-5p was observed in α-TC1 cells compared to β-TC3 cells, indicating that cytokine-independent factors expressed in α-cells induce a higher basal level of miR-146a-5p. Interestingly, the miR-146a-5p promoter also contains binding sites for OCT-1, which in Mutu cells have been shown to play a substantial role in facilitating constitutive miR-146a-5p expression [45], and the expression level of OCT-1 is higher in α-cells compared to β-cells based on publicly available array data (www.t1dbase.org). We found cytokine-induced miR-146a-5p expression to be more prominent in Pdx-1 overexpression-driven β-cell maturation. Since miR-146a-5p targets proximal IL-1 signaling components, a high basal expression level could contribute to the α-cell resistance to cytokines. Moreover, blunted IL-1β signaling in α-cells [54] may explain the impaired cytokine-induced miR-146a-5p expression in α- compared to β-cells.

miR-146a-5p is regulated by other mediators of β-cell dysfunction than cytokines, such as high glucose [43, 44] and palmitate [55]. miR-146a-5p was increased by 2.8-fold in islets of leptin-deficient ob/ob obese diabetes-resistant mice compared with lean mice, but only induced by 1.2-fold in diabetes-prone non-diabetic mice [56]. Islets from the non-obese diabetes (NOD) mouse model also expressed increased miR-146a-5p levels at disease-onset [43, 57], and miR-146a-5p was increased in the islets of adult diabetic mice [58]. Our observation that miR-146a-5p was elevated in diabetes-prone BB-DP rat pancreas at diabetes onset strengthens the body of evidence for the potential role of miR-146a-5p in β-cell death and T1D.

Cytokines induce miR-146a-5p expression in human monocytes [34], and the expression of miR-146a-5p is dysregulated in peripheral blood mononuclear cells from T1D patients [59]. Moreover, miR-146a-5p-deficient mice develop a severe myeloproliferative syndrome, leading to autoimmune or inflammatory diseases before 24 weeks of age [60]. Taken together with evidence discussed in the preceding paragraphs, these observations support a protective role for miR-146a-5p against inflammatory and autoimmune mechanisms leading to diabetes. It is gratifying that our unbiased array approach in INS1 cells identified the importance of this miR, underpinning the strength of an approach using interventions, i.e. KDACi, to reveal novel candidate miRs of therapeutic potential. Future studies should pay attention also to other miRs modulated by KDACi in INS1 cells and confirm their expression in human islets as well, and such work is in progress.

In conclusion, our data together with the majority of the literature point towards a protective role of miR-146a-5p against cytokine-induced β-cell apoptosis and suggest that long-term expression of miR-146a-5p can be enhanced by KDACi via epigenetic modification. The study by Roggli *et al*. suggested a pro-apoptotic effect of miR-146a-5p in β-cells [43]. This apparent controversy illustrates the complexity of miR biology, where the duration and the intensity of miR-146a-5p overexpression may differently impact β-cell function and survival, and where the kinetics and inflammatory context of changes in miR-146a-5p levels may determine if miR-146a-5p attenuates inflammation or favors apoptosis. Thus, the role of miR-146a-5p as a pathogenetic or a protective factor in diabetes is yet not finally settled. It remains to be investigated whether the miR-146a-5p expression level in β-cells is adequate to constitute an efficient apoptosis brake *in vivo*. Antagomirs, either in the form of locked nucleic acids (LNAs) or short oligonucleotides, have shown activity *in vivo* in disease models e.g. of genetic hypercholesterolemia [61, 62]; anti-miR-146a-5p antagomirs should be tested in diabetic animal models to evaluate the translational potential of our findings.

## Supporting information

S1 Fig KDACi reduced cytokine-induced apoptosis and NO accumulation in INS1 cells. INS1 cells were left untreated (-), treated with 1 μM CI994 or 125 nM Givinostat (Giv.) 1 h prior to addition of 0.1 ng/mL IFN-γ plus 37–150 pg/mL IL-1β (as indicated) or no cytokines (Ctrl) for 24 h. (A) Apoptosis as % of control measured with the Cell Death Detection ELISA from Roche. Data are presented as mean ± SEM (n = 4–10). (B) The media were analyzed for nitrite using the NO assay. Data are presented as mean ± SEM (n = 3–11). Statistical significance of paired t-test is shown in the graphs (*p<0.05, **p<0.01, ***p<0.001 and ****p<0.0001).
(TIF)

S2 Fig Target prediction and regulatory interaction networks of identified miR groups. The figure depicts regulatory interaction networks (RINs) of miR-targets for group A (S2A), group B (S2B) and group’s c and d (S2C). Each RIN includes two types of nodes: the miRs (cyan) and their predicted targets (pink) as identified from miRTarBase and TargetScan databases. The color of the connecting arrows for each RIN represents the two databases: miRTarBase (blue) and TargetScan (red).
(TIF)

S3 Fig Cytokine-induced miR-146a-5p expression in rat islets. (A) The miR-146a-5p expression was analyzed by qRT-PCR analysis in isolated rat islets exposed to IL-1β (160 pg/ml) or a combination of IL-1β (160 pg/ml) and IFN-γ (5 ng/ml). The data is presented as the mean of two experiments. The miR-146a-5p data was normalized to the internal control, let-7c. (B) Expression of let-7c treated with IL-1β (160 pg/ml) and a mix of IL-1β (160 pg/ml) and IFN-γ (5 ng/ml) for 24 h is stable.
(TIF)

S4 Fig miR-146a-5p targets TRAF6 and IRAK1 in INS1 cells. (A) Representative Western blot of iNOS, TRAF6, IRAK1 and β-actin (n = 4). INS1 cells were transiently transfected with a control oligo, miR-146a-5p, or anti-anti-miR-146a-5p oligo for 48 h, and exposed to media with or without IL-1β (160 pg/ml) for 6 h. (B) The luciferase assay was performed in INS1 cells transfected with luciferase gene and native 3’UTR constructs of TRAF6 together with control oligo, miR-146a-5p, or anti-miR-146a-5p oligo 24 h prior to harvest. Means ± SEM (n = 4). (C) INS1 cells were transfected with control oligo or miR-146a-5p for 48 h hours prior to RNA extraction, and mRNA levels of *TRAF6* normalized to *PPIA* levels were determined by qRT-PCR. Means ± SEM (n = 3). (D) INS1 cells were transfected with luciferase gene and native 3’UTR constructs of IRAK1 together with control oligo, miR-146a-5p, or anti-miR-146a-5p oligo 24 h prior to harvest. Means ± SEM (n = 4). (E) INS1 cells were transfected with control oligo or miR-146a-5p for 48 h prior to RNA extraction, and mRNA levels of *IRAK1* normalized to *PPIA* levels were determined by qRT-PCR. Means ± SEM (n = 3). *p<0.05.
(TIF)

S1 Table Functional annotation clustering of miR-targets from the selected four groups. The clustering of gene ontology (GO) biological process (BP) terms was performed in DAVID. Representative biological terms associated for each enriched cluster (group enrichment score > 1.3) are shown along with total number of genes in each cluster (Count) and gene names (Genes).
(DOCX)

S2 Table Two-way ANOVA test statistics of qRT-PCR, apoptosis and NO results. (DOCX)
