# Supplementary figures and images for: MicroRNAs and histone deacetylase inhibition-mediated protection against inflammatory β-cell damage
Source: PLoS One. 2018 Sep 27;13(9):e0203713. doi: 10.1371/journal.pone.0203713 (PMC6160007; doi:10.1371/journal.pone.0203713)

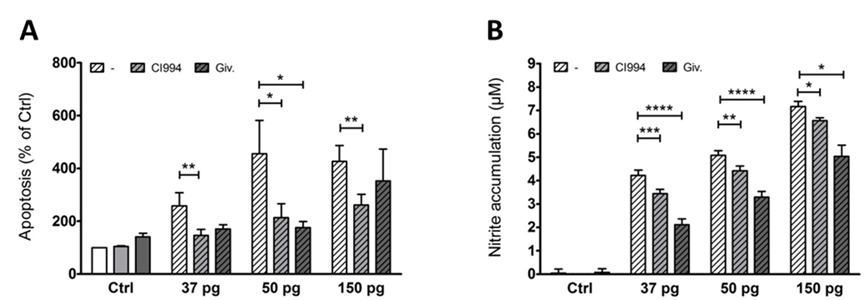

Supplement: S1 Fig — INS1 cells were left untreated (-), treated with 1 μM CI994 or 125 nM Givinostat (Giv.) 1 h prior to addition of 0.1 ng/mL IFN-γ plus 37–150 pg/mL IL-1β (as indicated) or no cytokines (Ctrl) for 24 h. (A) Apoptosis as % of control measured with the Cell Death Detection ELISA from Roche. Data are presented as mean ± SEM (n = 4–10). (B) The media were analyzed for nitrite using the NO assay. Data are presented as mean ± SEM (n = 3–11). Statistical significance of paired t-test is shown in the graphs (*p<0.05, **p<0.01, ***p<0.001 and ****p<0.0001). (TIF) [file pone.0203713.s001.tif]

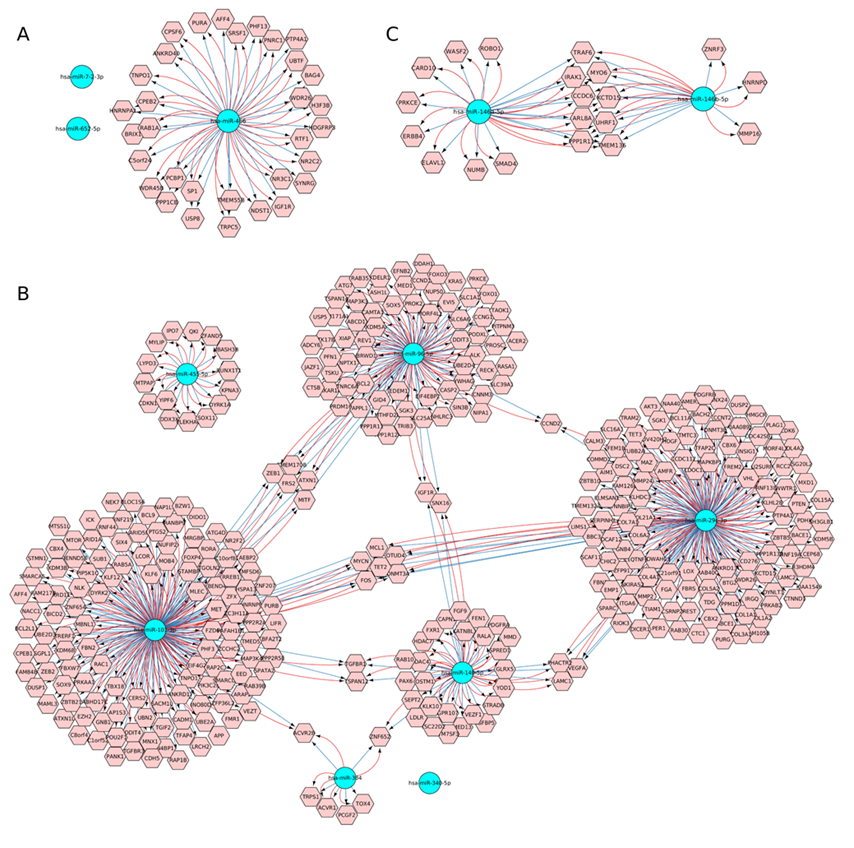

Supplement: S2 Fig — The figure depicts regulatory interaction networks (RINs) of miR-targets for group A (S2A), group B (S2B) and group’s c and d (S2C). Each RIN includes two types of nodes: the miRs (cyan) and their predicted targets (pink) as identified from miRTarBase and TargetScan databases. The color of the connecting arrows for each RIN represents the two databases: miRTarBase (blue) and TargetScan (red). (TIF) [file pone.0203713.s002.tif]

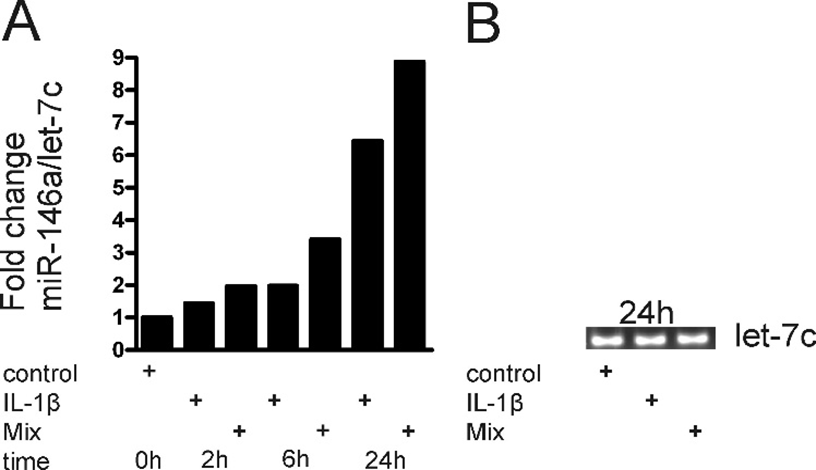

Supplement: S3 Fig — (A) The miR-146a-5p expression was analyzed by qRT-PCR analysis in isolated rat islets exposed to IL-1β (160 pg/ml) or a combination of IL-1β (160 pg/ml) and IFN-γ (5 ng/ml). The data is presented as the mean of two experiments. The miR-146a-5p data was normalized to the internal control, let-7c. (B) Expression of let-7c treated with IL-1β (160 pg/ml) and a mix of IL-1β (160 pg/ml) and IFN-γ (5 ng/ml) for 24 h is stable. (TIF) [file pone.0203713.s003.tif]

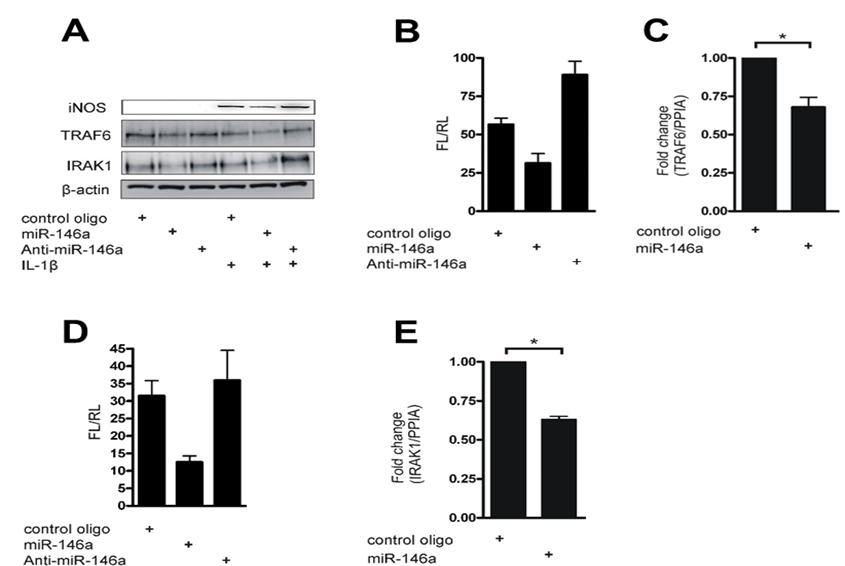

Supplement: S4 Fig — (A) Representative Western blot of iNOS, TRAF6, IRAK1 and β-actin (n = 4). INS1 cells were transiently transfected with a control oligo, miR-146a-5p, or anti-anti-miR-146a-5p oligo for 48 h, and exposed to media with or without IL-1β (160 pg/ml) for 6 h. (B) The luciferase assay was performed in INS1 cells transfected with luciferase gene and native 3’UTR constructs of TRAF6 together with control oligo, miR-146a-5p, or anti-miR-146a-5p oligo 24 h prior to harvest. Means ± SEM (n = 4). (C) INS1 cells were transfected with control oligo or miR-146a-5p for 48 h hours prior to RNA extraction, and mRNA levels of TRAF6 normalized to PPIA levels were determined by qRT-PCR. Means ± SEM (n = 3). (D) INS1 cells were transfected with luciferase gene and native 3’UTR constructs of IRAK1 together with control oligo, miR-146a-5p, or anti-miR-146a-5p oligo 24 h prior to harvest. Means ± SEM (n = 4). (E) INS1 cells were transfected with control oligo or miR-146a-5p for 48 h prior to RNA extraction, and mRNA levels of IRAK1 normalized to PPIA levels were determined by qRT-PCR. Means ± SEM (n = 3). *p<0.05. (TIF) [file pone.0203713.s004.tif]
