# Supplementary material for: MicroRNAs and histone deacetylase inhibition-mediated protection against inflammatory β-cell damage
Source: PLoS One. 2018 Sep 27;13(9):e0203713. doi: 10.1371/journal.pone.0203713 (PMC6160007; doi:10.1371/journal.pone.0203713)
Supplement: S1 Table — The clustering of gene ontology (GO) biological process (BP) terms was performed in DAVID. Representative biological terms associated for each enriched cluster (group enrichment score > 1.3) are shown along with total number of genes in each cluster (Count) and gene names (Genes). (DOCX) [file pone.0203713.s005.docx]

| GO BP Clusters | Representative Term | Enrichment Score | Count | Genes |
| --- | --- | --- | --- | --- |
| Group A | | | | |
| No enriched clusters | | | | |
| Group B | | | | |
| Cluster1 | Negative regulation of transcription | 6.85 | 32 | CBX4, CBX2, ZEB2, ZEB1, REST, SOX9, PRDM16, CBFA2T2, PCGF2, SIN3B, POU2F1, EED, PER1, NR2F2, KDM5B, DNMT3B, DNMT3A, NACC1, KLF12, VHL, ARID5B, WWTR1, FOXP4, PURB, ATXN1, CTNNBIP1, HDAC4, CDKN1B, TRPS1, JAZF1, LCOR, KDM6B |
| Cluster2 | Regulation of phosphorylation | 6.66 | 31 | CCNT2, HMGCR, ADCY6, TRIB3, ZEB2, CCNG1, PTEN, ZFP91, APP, MAP3K4, KRAS, BCL2, RAC1, PRKAA1, SPRED1, FRS2, TGFBR1, MET, STRADB, PRKCE, ATXN1, PROK2, ACVR2B, CCND1, YWHAG, DUSP2, CDKN1B, CCND2, PRKAR1A, PDGFRB, MTOR |
| Cluster3 | Regulation of apoptosis | 6.38 | 42 | CADM1, PTGS2, MCL1, XIAP, MITF, CBX4, STK17B, FOXO1, FOXO3, SOX9, FEM1B, PTEN, ZFP91, IGF1R, PCGF2, APP, KRAS, TIAM1, SH3GLB1, BCL2, RAC1, DYRK2, CASP2, RASA1, STAMBP, NACC1, SGK3, VHL, TGFBR1, STRADB, PRKCE, DDIT3, BCL2L11, PROK2, CDKN1B, BTG2, DUSP1, BBC3, VEGFA, PPP1R13B, CTSB, ACVR1 |
| Cluster4 | Negative regulation of cell death | 4.89 | 23 | STAMBP, SGK3, MCL1, XIAP, VHL, TGFBR1, MITF, CBX4, FOXO1, STRADB, PTEN, ZFP91, PROK2, IGF1R, PCGF2, KRAS, BTG2, SH3GLB1, BCL2, VEGFA, CASP2, RASA1, ACVR1 |
| Cluster5 | Cell migration | 4.54 | 20 | SGPL1, ZFAND5, HMGCR, VHL, PODXL, ARID5B, TGFBR1, MET, PAX6, ZEB2, SIX4, PTEN, ITGA6, RAC1, PDGFRB, TGFBR3, PAFAH1B1, LAMC1, NR2F2, ACVR1 |
| Cluster6 | Positive regulation of cell death | 3.25 | 22 | NACC1, CADM1, PTGS2, TGFBR1, STK17B, FOXO3, PRKCE, FEM1B, PTEN, DDIT3, BCL2L11, APP, CDKN1B, DUSP1, BBC3, SH3GLB1, TIAM1, BCL2, RAC1, PPP1R13B, DYRK2, CASP2 |
| Cluster7 | Collagen metabolic process | 2.32 | 5 | TRAM2, COL3A1, COL1A1, SERPINH1, MMP2 |
| Group C and D | | | | |
| Cluster1 | NF-κB-inducing kinase activity | 2.96 | 3 | IRAK1, TRAF6, CARD10 |
| Cluster2 | regulation of phosphorylation | 2.81 | 5 | IRAK1, SMAD4, TRAF6, PRKCE, CARD10 |
| Cluster3 | Regulation of cell development | 2.38 | 3 | ROBO1, NUMB, SMAD4 |
