# Supplementary material for: MicroRNAs and histone deacetylase inhibition-mediated protection against inflammatory β-cell damage
Source: PLoS One. 2018 Sep 27;13(9):e0203713. doi: 10.1371/journal.pone.0203713 (PMC6160007; doi:10.1371/journal.pone.0203713)
Supplement: S2 Table — (DOCX) [file pone.0203713.s006.docx]

| **miR-7a-2-3p** | **P-value** |
| --- | --- |
| Interaction | 0.0419 |
| Treatment | < 0.0001 |
| Time | 0.4427 |
| **miR-101a-3p** | **P-value** |
| Interaction | 0.1349 |
| Treatment | < 0.0001 |
| Time | 0.0046 |
| **miR-455-5p** | **P-value** |
| Interaction | 0.9746 |
| Treatment | 0.0324 |
| Time | < 0.0001 |
| **miR-29c-3p** | **P-value** |
| Interaction | 0.9124 |
| Treatment | 0.0242 |
| Time | 0.0487 |
| **miR-340-5p** | **P-value** |
| Interaction | 0.9778 |
| Treatment | 0.0239 |
| Time | 0.4410 |
| **miR-146b-5p** | **P-value** |
| Interaction | 0.3303 |
| Treatment | < 0.0001 |
| Time | 0.0257 |
| **miR-96-5p** | **P-value** |
| Interaction | 0.9839 |
| Treatment | 0.5248 |
| Time | 0.0190 |
| **miR-384-5p** | **P-value** |
| Interaction | 0.9784 |
| Treatment | 0.3382 |
| Time | 0.1583 |
| **miR-146a-5p** | **P-value** |
| Interaction | < 0.0001 |
| Treatment | < 0.0001 |
| Time | < 0.0001 |
| **Cell Death** | **P-value** |
| Interaction | 0.4862 |
| Treatment | 0.0046 |
| Cytokine dose | 0.0259 |
| **NO Assay** | **P-value** |
| Interaction | 0.9316 |
| Treatment | < 0.0001 |
| Cytokine dose | < 0.0001 |
